# Epidemiology, Pathophysiology, and Management of Cancer-Associated Ischemic Stroke

**DOI:** 10.3390/cancers16234016

**Published:** 2024-11-29

**Authors:** Dylan Ryan, Tarek Bou Dargham, Salman Ikramuddin, Shashank Shekhar, Soma Sengupta, Wuwei Feng

**Affiliations:** 1 Department of Neurology, Duke University School of Medicine, Durham, NC 27704, USA; shashank.shekhar@duke.edu (S.S.); wayne.feng@duke.edu (W.F.); 2 Department of Neurosurgery, The Preston Robert Tisch Brain Tumor Center, Duke University Medical Center, Durham, NC 27710, USA; tarek.boudargham@duke.edu; 3 Department of Neurology, University of Texas Health Sciences Houston, Houston, TX 77030, USA; ikram005@umn.edu; 4 Department of Neurology, University of North Carolina, Chapel Hill, NC 27599, USA; ssengup@email.unc.edu

**Keywords:** stroke, cancer, ischemic stroke, epidemiology

## Abstract

Cancer and stroke are leading causes of global disability and mortality. With improvements in cancer-associated mortality and advancements in treatment of active malignancy, it is more common to encounter patients with ischemic stroke and active malignancy. Evidence suggests that cancer-associated ischemic stroke is a unique subtype of stroke; however, there is limited guidance when considering diagnostic workup, secondary prevention, rehabilitation, and future directions within this population. In this narrative review, we aim to describe the epidemiology, pathophysiological mechanisms, management, and future directions regarding understanding of cancer-associated ischemic stroke.

## Simple Summary

Cancer and stroke are leading causes of global disability and mortality. With improvements in cancer-associated mortality and advancements in treatment of active malignancy, it is more common to encounter patients with ischemic stroke and active malignancy. In this paper, we first review the epidemiology regarding cancer-associated ischemic stroke and management challenges. We then highlight the pathophysiological contributors to the development of hypercoagulability, as well as tumor-associated and treatment-associated contributors to stroke in patients with cancer. The final section addresses acute treatment, secondary prevention, recovery, and future directions regarding management of cancer-associated stroke.

## 1. Introduction

Both cancer and stroke are leading causes of global disability and mortality [1]. Concomitant cerebrovascular disease is common amongst certain types of cancer patients and is a significant cause of further disability and mortality [2], which can impact overall treatment and prognosis within this vulnerable but expanding population. Cerebrovascular risk factors and disease can increase the risk of stroke themselves, but active cancer can also cause stroke via a variety of mechanisms, including hypercoagulability, paradoxical embolization, tumor embolism, non-bacterial endocarditis, direct tumor compression, and complications from chemotherapy. With cancer mortality declining and more cancer patients surviving from advanced treatment [3], it is further paramount to understand the prevention and treatment of cancer-associated ischemic stroke (CAIS) to prevent long-term morbidity and mortality. The incidence of cancer-associated thrombosis has been found to be increasing over time, with highest rates in pancreatic, brain, lung, and ovarian cancer and lowest in prostate and breast cancer [4].

Although cancer is emerging as a common risk factor for all-cause stroke, there remain limited clinical practice guidelines and directed recommendations regarding stroke prevention and therapy within this population [5,6]. Given the increasing incidence of cancer worldwide, it is of further importance to better equip medical providers with an understanding of CAIS to aid in decision-making. Several unique risk factors as well as treatment considerations exist when treating patients with active cancer that create challenges for neurologists and oncologists when treating this subgroup of patients.

The aim of this narrative review is to comprehensively examine epidemiology, pathophysiology, prevention, treatment, recovery, and future directions regarding CAIS.

## 2. Epidemiology

Cerebrovascular disease is common in cancer patients, with approximately 15% of cancer patients suffering from cerebrovascular disease [7]. A study of autopsies has previously shown that the most common central nervous system complication of cancer is cerebral infarction, with 14.6% of patients having pathological evidence of cerebrovascular disease [8]. Cancer and stroke share many risk factors, including obesity, smoking, and atherosclerotic disease [9]. Upwards of 13% of patients with ischemic stroke have a known history of cancer [10], with the risk of ischemic events being higher in patients with cancer as compared to matched controls [11]. Stroke can even be the initial presentation of cancer, with 2–10% of patients diagnosed with cryptogenic stroke subsequently diagnosed with cancer within one year of stroke [12].

The risk of stroke in patients with cancer is highest amongst patients with distant metastases and within six months of diagnosis [12]. The risk of stroke appears to correlate with the aggressiveness of the cancer itself, as metastatic lung, pancreatic, and colorectal cancers appear to carry the highest risk [13]. These cancer types were also most highly associated with the diagnosis of cancer post-incident stroke [14]. Cancers most highly associated with venous thromboembolism (VTE) have also been most highly associated with stroke, specifically lung and pancreatic cancer [13], speaking to underlying hypercoagulability.

In approximately half of cancer patients diagnosed with ischemic stroke, the etiology of ischemic stroke remains undetermined after standard diagnostic imaging and evaluation [12], with the estimated proportion of embolic stroke of undetermined source (ESUS) patients with cancer approaching 10%, although it may be as high as 20% in some Asian populations [15]. Recurrent stroke rates in patients with ESUS and cancer within one year are as high as 29% [12]. Within this population, diagnosing an etiology could potentially impact overall treatment decisions regarding secondary stroke prevention. Aspects of workup that can hint at the presence of occult cancer include elevated D-dimer, elevated C-reactive protein, anemia, history of smoking, unexplained weight loss, and infarcts in all vascular distributions [12]. Though the presence of findings such as the three-territory sign is common, the yield of transthoracic echocardiography (TTE) is low within this population [16], signifying the challenges in diagnosing an underlying etiology within this population. There further remains uncertainty regarding cancer screening and whole-body imaging in patients meeting ESUS criteria. Prospective data assessing advanced imaging used for cancer screening and diagnosis after unprovoked VTE have been of low diagnostic yield with no impact on overall mortality [17]. Elevated plasma D-dimer and ischemic lesions in multiple vascular territories have been found to be independent predictors of occult cancer in patients with cryptogenic stroke [18,19], though utilization of chest and abdominal imaging for cancer screening has unclear widespread effectiveness for cancer detection in this population. Fibrin monomer levels have been found to be a better indicator than plasma D-dimer in distinguishing patients with overtly disseminated intravascular coagulation [20], though utilization in patients with CAIS is unclear.

Further evidence has suggested that cancer-associated ischemic stroke, specifically embolic stroke, is a distinct subtype of stroke. There is not yet a reliable serum biomarker for cancer-associated ischemic stroke, though progress has been made regarding improving the diagnosis of cancer in patients with cryptogenic stroke. Patients with CAIS have been shown to have differential gene expression patterns as compared to stroke-only and cancer-only controls [21]. There has also been found to be specific miRNA-mRNA networks that differ between patients with CAIS as compared to stroke-only and cancer-only controls, and differential networks based on stroke etiology [22].

## 3. Pathophysiology

**Figure 1:**
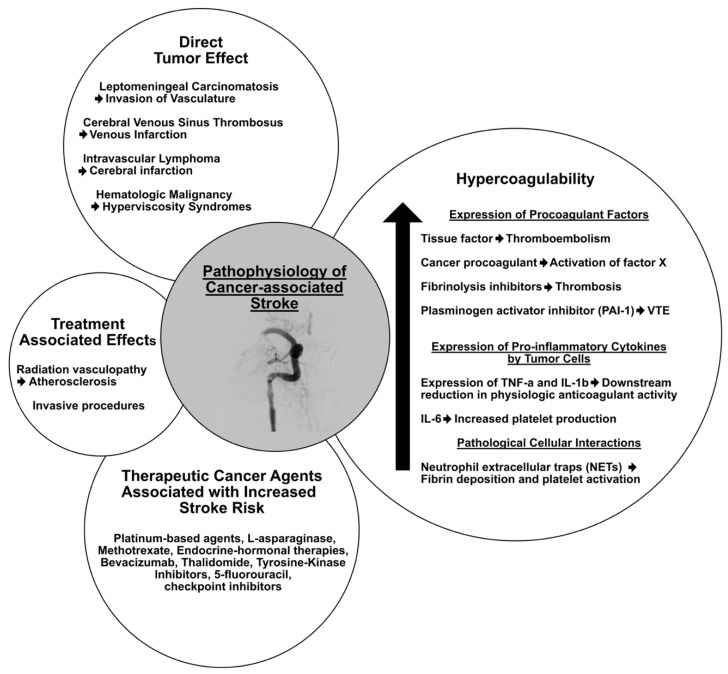
Pathophysiological mechanisms of cancer-associated ischemic stroke.

Several pathophysiological contributors exist when considering mechanisms regarding CAIS (Figure 1). These can relate to hypercoagulability, direct tumor-associated factors, and treatment related to the underlying malignancy. Establishing contributing mechanisms within this population is an important consideration regarding treatment decisions for oncologists and neurologists, though this can be challenging given multiple factors may be related to the cause of the underlying stroke.

## 4. Cancer-Associated Hypercoagulability or Cancer-Induced Hypercoagulable State

Cancer-associated hypercoagulability has long been observed given the association between cancer and VTE, though this state also carries an increased risk of arterial thrombosis [23]. The pathophysiology behind cancer-associated hypercoagulability is complex and involves several factors related to cancer pathology and histology, procoagulant factors, inflammatory cytokines, and cell–cell interactions.

The most notable of these procoagulant factors is tissue factor, which is expressed on normal endothelium and initiates the coagulation cascade [24]. Tumor cells can express high levels of tissue factor and can release extracellular vesicles used in cell–cell communication and for tumor growth that can further express tissue factor and increase the risk of thromboembolism [25]. These vesicles have been further shown to correlate with elevated D-dimer levels, and patients with cancer-associated ESUS have been shown to have higher levels of these circulating vesicles [26]. Tumor cells can also exhibit cancer procoagulant, which can independently activate factor X [27] and contribute to cancer-associated hypercoagulability. Cancer procoagulant has been found to be elevated in 85% of cancer patients [28], with a wide range of pathologies that includes lung, ovarian, breast, prostate, colorectal, and renal cancer [29], and is correlated with hypercoagulability.

Fibrinolysis inhibitors can also concurrently contribute to these procoagulant factors in leading to thrombosis. Tumor cells can express fibrinolytic regulators on their cell surface, which can contribute to tumor growth and spread but can further lead to vascular complications. Expression of plasminogen activator inhibitor-1 (PAI-1), a key regulator of plasminogen activation, has been shown to be correlated with risk of VTE, especially in pancreatic cancer [30]. Doubling levels of PAI-1 have been shown to increase the risk of VTE by 40% in this population [31].

Tumor cells also produce several proinflammatory cytokines that play a role in associated hypercoagulability. Expression of TNF-α and IL-1β can further induce expression of endothelial tissue factor [32], as well as downregulate expression of thrombomodulin [33]. This reduction in endothelial thrombomodulin leads to a reduction in physiologic anticoagulant activity [34], as this protein potentiates activity of protein C and protein S. Given that protein C and protein S act as potent anticoagulants, the downregulation of thrombomodulin contributes to the production of thrombin and thrombus formation. TNFR1 and TNFR2 have been found to be upregulated in CAIS [22], further contributing to the role inflammatory pathways play on hypercoagulability in cancer. IL-6, which increases platelet production through regulation of thrombopoietin, has also been shown to be elevated in CAIS [22]. Upregulation and release of vascular endothelial growth factor (VEGF) can also lead to multiple cell types increasing expression of tissue factor [35], which in turn can lead to further activation of procoagulant pathways. This is further correlated with findings linking tumor angiogenesis with hypercoagulability in patients with cancer [36]. These proinflammatory cytokines can further induce a procoagulant phenotype in endothelial cells [37] with further expression of tissue factor and PDI-1.

Cellular interactions also play a role in the development of hypercoagulability. Neutrophil extracellular traps (NETs), which are released from dying neutrophils, have been shown to be associated with an increased risk of VTE [38]. NETs can act as a substrate for fibrin deposition and platelet activation, and increasing levels of NETosis have been associated with CAIS [39]. Furthermore, platelets exhibit increased activation in patients with cancer [40] with increased levels of aggregation. ICAM1 and SELP, which encodes p-selectin, are associated with platelet activation and have been shown to be upregulated in CAIS [22]. Elevated soluble p-selectin has been shown to be associated with VTE in patients with cancer [41]. Analysis of arterial clots in patients with cancer have been shown to have high proportions of platelets [42,43], further suggesting that dysregulation of platelet activation plays an important role in cancer-associated hypercoagulability. Inflammatory cytokines can also lead to further circulation of monocytes and macrophages, which in cancer patients have significantly increased expression of tissue factor [44].

## 5. Direct Tumor Effects

Direct tumor invasion of the vasculature can be difficult to identify, but it can lead to the development of cerebral ischemia. Leptomeningeal carcinomatosis can lead to direct invasion of the vasculature, which can lead to local thrombosis and/or vasospasm leading to ischemia [45]. Occlusion of venous outflow tracts and hypercoagulable state can lead to cerebral venous sinus thrombosis (CVST), increasing the risk of venous infarction and hemorrhage in patients with CNS malignancy [46]. Rarely, tumor embolization can lead to ischemic infarction [47] and can even lead to aneurysmal formation that increases the risk of hemorrhage. Intravascular lymphoma, a rare presentation of CNS lymphoma with poor prognosis, can present with multifocal, diffuse infarcts [48]. Cardiac tumors, such as myxoma and fibroelastoma, can also lead to embolic stroke through local thrombosis and embolization or direct tumor embolization.

Hematologic malignancies illicit specific phenomena, such as hyperviscosity syndromes that can cause direct ischemia [49]. Expansion of a cell line or acellular components, such as increased protein levels, can lead to reduced flow that can directly lead to hypoperfusion and microcirculatory dysfunction. These expansions can also lead to increased erythrocyte aggregation and thus an increased propensity to form local thrombi. Acute myelogenous leukemia and chronic lymphocytic leukemia can lead to leukostasis and thus increase the risk of stroke [48]. In multiple myeloma, hyperviscosity can occur due to elevated protein levels. Bing–Neel syndrome, a rare complication of Waldenstrom macroglobulinemia, can lead to lymphoplasmacytic infiltration of cerebral vascular arteries, causing encephalopathy and stroke [50].

## 6. Treatment-Associated Effects

Several chemotherapies have been associated with increased risk of stroke in patients with cancer (Table 1). Given the effects of cancer on hypercoagulability and the association of aggressive malignancies with increased risk of stroke, the risks of these agents must be weighed against the overall risk that the cancer itself imposes.

Platinum-based chemotherapy regimens, such as those with cisplatin and carboplatin, have been associated with increased risk of stroke [48]. Infusions of platinum-based chemotherapy have been associated with increases in circulating endothelium-derived and platelet-derived microparticles that may increase the risk of stroke using these agents [51]. In patients with ovarian cancer, those treated with platinum-based regimens as compared to those on non-platinum-based regimens had a higher relative risk of stroke [52].

L-asparaginase, when used for hematologic malignancies, has been long associated with an increased risk of cerebral thrombosis [48]. Rates of ischemic stroke using this therapy are as high as 4.2% [53]. Patients using L-asparaginase need close neurologic monitoring given known cerebrovascular complications.

Methotrexate has been associated with ischemic stroke and impaired cerebral autoregulation [54]. Intrathecal use of methotrexate in children with CNS tumors has been associated with silent cerebrovascular disease and lacunar stroke [55]. Childhood survivors of CNS tumors with use of methotrexate were found to be 40 times more prone to stroke than their sibling controls [56], though the mechanism behind this association is unclear.

Endocrine hormonal therapies can also impact overall stroke risk. Raloxifene, a selective estrogen receptor modulator (SERM) used in the treatment of breast cancer, has been associated with a higher incidence of stroke [57]. Tamoxifen, the most commonly used SERM, has been shown to have associations with increased risk of stroke and venous thromboembolism [58]. Patients with prostate cancer treated with androgen deprivation therapy (ADT) have also been found to be at increased risk of stroke [59,60].

Bevacizumab, a VEGF inhibitor commonly used for treatment of cerebral edema and radiation necrosis in patients with glioma and certain brain metastases, has been associated with cerebral ischemia and hemorrhage [61]. It is also used as a treatment in metastatic colorectal carcinoma, with a meta-analysis suggesting increased risk of cerebral ischemic events when used in this population [61]. A meta-analysis showed bevacizumab to be associated with increased risk of ischemic stroke, especially in those treated with higher doses [62].

Lenalidomide and thalidomide are antiangiogenic, immunomodulatory agents used in the treatment of multiple myeloma. Both have been associated with increased risk of arterial and venous thromboembolic events [63]. Given this association, thromboprophylaxis is used with the use of these drugs [64].

There are multiple tyrosine kinase inhibitors, which are used in the treatment of chronic myeloid leukemia and acute lymphoblastic leukemia, that carry increased risk. A meta-analysis comparing ponatinib, nilotinib, and dasatinib as compared to imatinib showed increased risk of stroke and vascular occlusive events [65].

5-Fluorouracil (5-FU) is used in several chemotherapy regimens for a variety of cancers. Case series have suggested an association with stroke [66], which could be a consideration when used alongside platinum-based chemotherapies. 5-FU has also been associated with cerebral vasospasm [67], which can prevent stroke and stroke-like events. Though there has not been a conclusive association with an increased risk of stroke [68], awareness of these cases could aid future treatment decision-making.

Checkpoint inhibitors are now more frequently used in the treatment and maintenance therapy of a variety of cancers. These therapies have been associated with several neurologic complications that are often immune-mediated. This drug class has been associated with cases of vasculitis that can further increase the risk of stroke [69].

Erythropoiesis-stimulating agents, which can be used in patients with symptomatic anemia, have been associated with increased risk of thromboembolic events [70]. Drug labeling has noted increased risk of stroke with elevated hemoglobin targeting for patients with symptomatic anemia [68].

Although use of doxorubicin and anthracyclines is not directly linked to increased risk of stroke [68], induced cardiomyopathy could play a role in stroke pathophysiology in this population. Similarly, trastuzumab, used in HER2-positive breast cancer, is associated with an increase in cardiovascular events [71]. Knowledge of these complications can aid in workup and treatment decision-making between neurologists and oncologists.

Chimeric antigen receptor T-cell (CAR-T) therapy is being increasingly studied for use in several malignancies. CAR-T has been associated with a diagnosis of new VTE of up to 11% in patients within three months of therapy [72,73]. CAR-T therapy has been associated with a number of neurologic complications, including stroke. High CAR-T dose, acute lymphoblastic leukemia, and cytokine release syndrome have been associated with increased risk of neurologic adverse events with endothelial disruption, multifocal vascular disruption, and disseminated intravascular coagulation [74].

**Table 1:** Classification of cancer treatments and their associated stroke risks.

Treatment Type	Medication	Mechanism
Chemotherapy	Platinum-based (cisplatin and carboplatin) [48,51,52]	Associated with increases in circulating endothelial-derived and platelet-derived microparticles
	L-asparaginase [53,75]	Procoagulant activity
	Methotrexate [54,55,56]	Impaired cerebral autoregulation
	5-Flurouracil [66,67,68]	associated with cerebral vasospasm, which can present with stroke and stroke-like symptoms
	Anthracycline (Doxorubicin) [68]	Induced cardiomyopathy could potentially contribute to stroke risk
Hormonal therapy	Endocrine hormonal therapies (Raloxifene, Tamoxifen, and ADT) [57,58,59,60]	Alteration in coagulation factors, including promoting coagulation and impairing anticoagulation
Immunomodulatory agents	Lenalidomide and thalidomide [63,64]	Procoagulant activity
Immunotherapy	Checkpoint inhibitors [69]	Associated with cases of vasculitis which can further increase risk of stroke
	CAR-T therapy [72,73,74]	Cytokine release syndrome, immune effector cell-associated neurotoxicity
Targeted Therapy	Tyrosine kinase inhibitors (Ponatinib, Nilotinib, and Dasatinib) [65,76]	Hypertension, cardiac arrhythmias, hyperglycemia, promotion of atherosclerosis, and platelet dysfunction
	VEGF inhibitor (Bevacizumab) [61,62]	Increases inflammation, impairs endothelial function, reduces NO and prostacyclin, and raises blood viscosity due to excess erythropoietin.
	HER2 inhibitors (Trastuzumab) [69]	Induced cardiotoxicity
Other	Erythropoiesis-stimulating agents [68,70]	Risk for stroke when targeting hemoglobin levels higher than 12g/dl leading to hyper viscosity and higher blood pressure

Abbreviations: ADT: androgen deprivation therapy; CAR-T: chimeric antigen receptor T-cell; HER2: human epidermal growth factor receptor 2; VEGF: vascular endothelial growth factor.

Radiation to the head and neck can also lead to direct vascular complications. Radiation therapy can affect vessels of all caliber [77], causing accelerated atherosclerotic disease, vasospasm, local thrombosis, fistulization, microhemorrhages, and aneurysm formation [78]. Rates of carotid stenosis following external radiation to the neck are as high as 60% [79]. These effects can be both acute and chronic, leading to long-term damage that can increase the risk of stroke.

Invasive procedures, such as surgeries and chronic line placements, have also been associated with stroke in patients with cancer [12]. Surgery can lead to stroke through multiple mechanisms, such as inducing tumor emboli, peri-operative and post-operative arrhythmia, and arterial injury. Pulmonary interventions, such as those for lung cancer, have been associated with peri-operative and post-operative stroke [48]. In patients undergoing resection for glioma, there were high rates of infarct adjacent to the resection cavity [80]. Indwelling catheters and ports increase the risk of infection and must be monitored given the risk of endocarditis in patients as well.

## 7. Treatment

There remains clinical equipoise regarding acute stroke management in patients regarding acute management and secondary prevention. Patients with cancer are often excluded from clinical trials and given concerns regarding complications related to cancer, such as bleeding, and long-term functional status. Current evidence can aid in decision-making with these patients considering management of their stroke and cancer.

## 8. Acute Treatment

For patients presenting within the thrombolysis window with presentation consistent with acute ischemic stroke, there is limited evidence regarding treatment with intravenous thrombolysis (IVT). Contraindications for use currently exist in patients with intracranial tumors and gastrointestinal malignancy [81], as well as those with recent major bleeding, given the theoretical risk of harm within these populations. Use of IVT in patients with active malignancy does not have clear contraindications, and American Heart Association (AHA) guidelines note that use of IVT is reasonable in those with a prognosis greater than six months [81]. A systematic review and meta-analysis of the use of IVT in patients with cancer found no significant difference in efficacy as compared to non-cancer patients regarding 3-month mortality and in-hospital mortality, symptomatic intracerebral hemorrhage (sICH), hemorrhagic transformation (HT), and functional independence [82]. Within this same study, there was not found to be a difference between gastrointestinal malignancies and other malignancies [82], suggesting that gastrointestinal malignancy may not be an absolute contraindication to use of IVT. It is unlikely, given the overall efficacy of IVT, that a randomized clinical trial (RTC) will be performed regarding thrombolysis in patients with systemic malignancy, though this would be of clinical utility. Considering current evidence, for patients without other absolute contraindications, use of IVT in patients with cancer presenting with acute stroke appears safe and efficacious.

Patients with cancer have also largely been excluded from major trials assessing the use of endovascular thrombectomy (EVT) for large vessel occlusion (LVO). There is no clear contraindication for use of EVT in patients with cancer [81], though prognosis and functional status must be considered when discussing this therapy. In an analysis of the Italian Registry of Endovascular Treatment in Acute Stroke, there was no reported difference in successful reperfusion, sICH, or probability in achieving functional independence at three months between group A and group B once propensity matched [83]. Given the large effect size of EVT in patients with LVO, there is unlikely to be a RCT that compared medical management to EVT in patients with cancer [84]. In patients with cancer with good premorbid functional status, reasonable quality of life and prognosis, and with goals of care in line with aggressive management, EVT for LVO is likely to yield better chances at functional independence than medical management.

## 9. Workup Considerations

Given associated and concurrent risk factors of cancer and stroke, providers should follow standard guideline-driven practice [5,81]. Though cancer can lead to hypercoagulability, it should not be discounted that patients with cancer can carry traditional risk factors that could drive the risk of stroke irrespective of the diagnosis of cancer. Patients with CAIS who meet ESUS criteria do tend to have fewer traditional risk factors [85] than those without cancer. Given the high rates of patients with cancer who also have cryptogenic mechanisms based on this initial workup, additional workup considerations should be considered whose goals of care are in line with continuous workup [12].

Given the association of cancer-associated hypercoagulability and elevated D-dimer, in patients with cryptogenic mechanisms, obtaining serum D-dimer levels could be of diagnostic benefit [86]. Elevated D-dimer levels have been shown to correlate with mortality, and reversal of hypercoagulability with antithrombotic therapy has been shown to be of prognostic benefit [87]. In patients with suspected hypercoagulable mechanisms, TEE should be considered given higher for diagnostic nonbacterial thrombotic endocarditis and sources of paradoxical embolism [16]. In patients with evidence of shunt, such as with a PFO, evaluation of lower extremity and pelvic veins [88] would be of clinical utility to assess the possibility of paradoxical embolization given that this would lead to a clear direction in management. Given high rates of bilateral emboli in patients with cancer [12] and multiple hypercoagulable mechanisms, transcranial dopplers with emboli monitoring should be considered, as they may provide further information into the mechanism of stroke as well as antithrombotic decision-making. In patients with indwelling catheters and ports, infectious sources as well as the possibility of local thrombosis should be assessed.

## 10. Secondary Prevention

Guidelines are sparse on the topic of antithrombotic management in patients with CAIS beyond patients with concomitant atrial fibrillation [5]. There are cases where use of therapeutic anticoagulation is clearly favored as compared to antiplatelet agents. In patients with VTE and cancer, American Society of Hematology guidelines recommend use of injectable low-molecular-weight heparins (LMWHs) or use of oral anticoagulants [64]. In patients with nonbacterial thrombotic endocarditis, use of LMWH is recommended [89].

In patients who have had workup and the mechanism remains cryptogenic, there remains significant clinical equipoise regarding antithrombotic choice in patients with cancer. Multiple RCT’s assessing use of therapeutic anticoagulation in patients with ESUS have failed to show superiority of this strategy [90,91,92,93], though there has not been a specific RCT assessing superiority of this strategy in patients with cancer. In a subgroup analysis of patients with cancer in NAVIGATE ESUS [94], which compared rivaroxaban and aspirin in patients meeting ESUS criteria, there was not a significant difference in recurrent stroke between the agents in patients with cancer. Similarly, a post hoc analysis of patients with cancer in ARCADIA [95], a trial comparing apixaban and aspirin in a population with biomarkers of atrial cardiopathy, found no difference in the risk of major ischemic or hemorrhagic events. A retrospective, single-center study of 172 patients with active cancer and acute ischemic stroke found no difference in recurrence rate between patients treated with anticoagulation and antiplatelet agents [96].

These studies certainly do not rule out the possibility of benefit of therapeutic anticoagulation in patients with active cancer and cryptogenic stroke. Given hypercoagulable mechanisms of stroke in patients with cancer, there remain considerations of using empiric anticoagulation within this population. In a small study of 29 patients with serial D-dimer measurements, the use of anticoagulation was associated with a reduction in serum D-dimer [97]. Further, the prospective OASIS-Cancer study [87] found in patients with highest elevations in serum D-dimer, reduction with use of anticoagulation improved one-year survival. The TEACH trial [98] found that use of LMWH in this population as compared to aspirin was feasible and safe, though enrollment was hampered by patients being averse to daily injections, further signified by the high rate of crossover in the trial. The ENCHASE trial [99], a pilot study comparing enoxaparin and edoxaban in cancer patients meeting ESUS, found similar reduction rates in serum D-dimer and microembolic signals at 90 days.

Though these theoretical considerations exist, there does exist evidence suggesting potential benefits of antiplatelet agents. Platelet activation is a mechanism behind cancer-associated hypercoagulability [40], and evaluation of thrombi in stroke patients with cancer has been rich in platelets [42,43]. Evidence of differential expression of ICAM1 and SELP [22], the latter being a target of antiplatelet therapy, suggests potential benefit of antiplatelets in patients with CAIS. Considering studies showing lack of clear superiority of therapeutic anticoagulation in patients with ESUS and no differences in subgroup analyses assessing recurrent stroke rate in patients treated with antiplatelets versus anticoagulants, it appears reasonable to treat with antiplatelet monotherapy rather than empiric anticoagulation.

Considering the above-mentioned information, there remain questions over the preferred antithrombotic strategy when approaching these patients. In patients where there is high suspicion of a hypercoagulable mechanism, that is, in those with the highest elevations in serum D-dimer, tumors associated with high rates of VTE, and positive microembolic signals, therapeutic anticoagulation with LMWH or oral anticoagulation should be considered. This must be weighed against the risk of bleeding given the high risk of major bleeding in cancer patients using anticoagulation [100]. Currently, with the lack of an RCT addressing anticoagulation versus antiplatelet in cryptogenic stroke in patients with cancer, risk–benefit discussions must be performed between patients and providers when considering antithrombotic choice within this population.

Aside from antithrombotic management, management of traditional vascular risk factors should also be employed. In patients with evidence of atherosclerotic disease, management with high-intensity statins is a guideline recommended for stroke secondary prevention [5]. Hyperlipidemia has been shown to be associated with stroke in patients after cranial radiation [101], and there is evidence that statin therapy may reduce the risk of stroke in patients after thoracic, cervical, and cranial radiation therapy [102]. Guideline-directed management of hypertension and diabetes, given their association with stroke, is also recommended within this population. In patients with PFO without evidence of associated VTE, PFO closure in eligible patients with good life expectancy regarding their cancer prognosis should be considered for given stroke risk reduction [103,104]. In patients where their chemotherapeutic is felt to be related to their stroke, this should be further discussed with their primary oncologist to determine the risks and benefits of continuing treatment, as well as alternative options.

Beyond antithrombotics and management of vascular risk factors, treatment of the underlying cancer is vital in trying to reduce recurrent thromboembolic events. In patients with cardiac tumors, resection of the tumor should be considered with the goal of reducing recurrent stroke [5]. Given active and metastatic cancer are associated with the development of hypercoagulability, treatment of the underlying cancer may be the most effective strategy in normalizing the hypercoagulable state and reducing subsequent stroke. In patients with myeloproliferative neoplasms, use of cytoreductive chemotherapy has been shown to reduce the risk of stroke and other vascular complications [105]. Challenges incorporating further treatment can be impacted by stroke-related disability, as stroke unfortunately may lead patients to no longer be candidates for further therapy given worsening functional status from stroke. Assessing the patient’s Karnofsky Performance Scale (KPS) could be of use to aid in discussing medical treatment and goals of care with the patient in regards to long-term prognosis and survival. In patients with adequate functional status who are potential candidates for continued treatment, use of directed chemotherapy with antithrombotics should be utilized in patients if this is in line with their goals of care.

## 11. Recovery

Considering the declining rate of stroke mortality and improvements in acute stroke management, there remains a growing population of patients with post-stroke disabilities requiring rehabilitation services. It is known that multidisciplinary, interprofessional stroke care that utilizes rehabilitative services improves functional outcome and independence post-stroke [106]. Patients discharged to inpatient rehabilitation facilities have been shown to have higher rates of return to community living as compared to skilled nursing facilities, though these patients historically have been younger patients with fewer comorbidities and post-stroke disability [107]. There have been several growing avenues in research, such as the use of neuromodulation and robotics for stroke recovery, though there remains limited evidence regarding stroke recovery in patients with cancer.

Patients with cancer are often excluded from trials and studies assessing the utilization of a variety of recovery services post-stroke. Decision-making in patients with cancer regarding utilization of rehabilitation services also does differ as compared to patients without cancer considering treatment. Long-term rehabilitation stays can impede access to cancer-related therapies, such as chemotherapy and radiation, that could aid in treating the underlying cancer. When considering discharge to a rehabilitation facility, providers must weigh the patient’s access to goal, and underlying goals related to their cancer. If the patient remains a candidate for cancer-related care given their underlying performance status post-stroke, then it is possible that utilization of at-home health services to allow access to not potentially delay cancer-related care should be considered. In patients where recovery could potentially improve functional status to meet their goals toward continued care for their cancer, then utilization of inpatient rehabilitation facilities could potentially be of higher benefit. It is paramount in this setting to have goals of care discussions with patients with CAIS to work toward comprehensive discharge plans that best meet the patient’s needs and are in line with their treatment goals.

## 12. Future Directions

As patients with cancer are more likely to survive from advanced chemotherapy, it can be expected that providers encountering patients with cancer and stroke will increase over time. Given this, there is an urgent need for future studies assessing optimal treatment strategies in these patients considering the underlying pathogenesis of stroke. Planning for an RCT comparing apixaban to aspirin in patients meeting ESUS criteria known as TEACH2 [12] is currently underway. A goal of TEACH2 is to use composite outcomes that include all major thromboembolic events to better capture the impact on quality of life and survival that these decisions entail. This trial would provide further evidence that holistically evaluates utilization of DOACs in patients with CAIS. There remains significant interest in this clinical question among stroke patients, given the role of hypercoagulability in the pathogenesis of stroke in patients with cancer.

Beyond questions of anticoagulation or antiplatelet monotherapy, there have been multiple recent studies employing a dual therapy approach in patients with conditions such as heart failure and atherosclerotic disease. Given that cancer-associated hypercoagulability involves platelet activation and activation of the coagulation cascade, a combination approach could potentially show clinical utility. Concerns regarding this approach would include higher rates of bleeding in patients with cancer that could mitigate potential benefits. These questions further illustrate the need for RCTs to assess antithrombotic management in patients with CAIS.

Given the high rates of cryptogenic stroke in patients with cancer and the elevated rates of patients with cryptogenic stroke later being diagnosed with cancer [10,12], there is a need to further elucidate stroke etiology in these cancers. Current workup, including body imaging and echocardiography, has been limited in further differentiating etiology [16,17,108,109]. Evidence of differential gene expression in patients with CAIS [21] and in cardioembolic versus noncardioembolic CAIS [22] could aid in developing serum biomarkers beyond serum D-dimer that could differentiate etiologies in patients with CAIS, such as with evaluation of fibrin monomer. These differentiations have the potential to aid in therapeutic decision-making regarding antithrombotic management in patients with CAIS with the goal of reducing recurrent thromboembolic events and reducing mortality. Larger data sets given the heterogeneity within this population and validation studies are needed, but these findings are promising when considering the potential impact on diagnosis and management. The MOST-Cancer study continues to investigate potential serum biomarkers [12] to aid in treatment decision-making regarding antithrombotic management in CAIS.

Beyond antithrombotic management, treatment considerations regarding management of the underlying cancer and the complications of treatment deserve further study. As the development of targeted therapies for a variety of solid and hematologic malignancies is developed, there will be a need to evaluate the role of these therapies in the risk reduction in stroke. As previously noted, treatment of the underlying malignancy could potentially be the most effective strategy in primary stroke prevention within this population given the impact on the underlying pathophysiology. Given the observed association of radiation necrosis and stroke [101], further study of the prevention of the complications of radiation as well as evaluation of therapies beyond antithrombotics could aid understanding and reducing stroke within this population.

## 13. Conclusions

CAIS is a unique subpopulation of patients that can be expected to grow as cancer survival continues to improve. Multiple pathophysiological mechanisms exist within this population that increase the risk of stroke and present unique challenges for neurologists and oncologists. Expanding traditional stroke workup to include serum D-dimer, TEE, and transcranial Doppler with microemboli detection could aid in elucidating hypercoagulable mechanisms to aid in patient-centric decision-making regarding antithrombotic management within this population. There is a need for continued collaboration of neurologists and oncologists to further identify and develop biomarkers that can aid in etiologic determination, as well as in the development of RCTs that aid in the understanding of antithrombotic choice and cancer-directed therapy within this population. Promising work utilizing miRNA and mRNA profiles could aid in further differentiating stroke mechanisms and etiologies to aid clinicians in treatment decisions. Through multidisciplinary collaboration, conducting studies to address the many open questions in CAIS could aid in the development of guideline-based practices that improve morbidity and mortality in this patient population.
